# Hydrogen Sulfide Plays a Key Role in the Inhibitory Neurotransmission to the Pig Intravesical Ureter

**DOI:** 10.1371/journal.pone.0113580

**Published:** 2014-11-21

**Authors:** Vítor S. Fernandes, Ana S. F. Ribeiro, Pilar Martínez, María Elvira López-Oliva, María Victoria Barahona, Luis M. Orensanz, Ana Martínez-Sáenz, Paz Recio, Sara Benedito, Salvador Bustamante, Albino García-Sacristán, Dolores Prieto, Medardo Hernández

**Affiliations:** 1 Departamento de Fisiología, Facultad de Farmacia, Universidad Complutense de Madrid, Madrid, Spain; 2 Departamento de Anatomía y Anatomía Patológica Comparadas, Facultad de Veterinaria, Universidad Complutense de Madrid, Madrid, Spain; 3 Departamento de Toxicología y Farmacología, Facultad de Veterinaria, Universidad Complutense de Madrid, Madrid, Spain; 4 Departamento de Investigación, Hospital Universitario Ramón y Cajal, Madrid, Spain; 5 Departamento de Urología, Hospital Universitario Puerta de Hierro-Majadahonda, Madrid, Spain; Cinvestav-IPN, Mexico

## Abstract

According to previous observations nitric oxide (NO), as well as an unknown nature mediator are involved in the inhibitory neurotransmission to the intravesical ureter. This study investigates the hydrogen sulfide (H_2_S) role in the neurogenic relaxation of the pig intravesical ureter. We have performed western blot and immunohistochemistry to study the expression of the H_2_S synthesis enzymes cystathionine γ-lyase (CSE) and cystathionine β-synthase (CBS), measurement of enzymatic production of H_2_S and myographic studies for isometric force recording. Immunohistochemical assays showed a high CSE expression in the intravesical ureter muscular layer, as well as a strong CSE-immunoreactivity within nerve fibres distributed along smooth muscle bundles. CBS expression, however, was not consistently observed. On ureteral strips precontracted with thromboxane A_2_ analogue U46619, electrical field stimulation (EFS) and the H_2_S donor *P*-(4-methoxyphenyl)-*P*-4-morpholinylphosphinodithioic acid (GYY4137) evoked frequency- and concentration-dependent relaxations. CSE inhibition with DL-propargylglycine (PPG) reduced EFS-elicited responses and a combined blockade of both CSE and NO synthase (NOS) with, respectively, PPG and N^G^-nitro-L-arginine (L-NOARG), greatly reduced such relaxations. Endogenous H_2_S production rate was reduced by PPG, rescued by addition of GYY4137 and was not changed by L-NOARG. EFS and GYY4137 relaxations were also reduced by capsaicin-sensitive primary afferents (CSPA) desensitization with capsaicin and blockade of ATP-dependent K^+^ (K_ATP_) channels, transient receptor potential A1 (TRPA_1_), transient receptor potential vanilloid 1 (TRPV_1_), vasoactive intestinal peptide/pituitary adenylyl cyclase-activating polypeptide (VIP/PACAP) and calcitonin gene-related peptide (CGRP) receptors with glibenclamide, HC030031, AMG9810, PACAP_6–38_ and CGRP_8–37_, respectively. These results suggest that H_2_S, synthesized by CSE, is involved in the inhibitory neurotransmission to the pig intravesical ureter, through an NO-independent pathway, producing smooth muscle relaxation via K_ATP_ channel activation. H_2_S also promotes the release of inhibitory neuropeptides, as PACAP 38 and/or CGRP from CSPA through TRPA_1_, TRPV_1_ and related ion channel activation.

## Introduction

Hydrogen sulfide (H_2_S) is considered as the third endogenous gaseous transmitter besides nitric oxide (NO) and carbon monoxide (CO) [1],[2]. H_2_S is synthesized from L-cysteine by the action of two pyridoxal-5′-phosphate-dependent enzymes, cystathionine γ-lyase (CSE) or cystathionine β-synthase (CBS) [1]–[5]. CBS activity is predominant in H_2_S synthesis in the central nervous system whereas CSE is the major H_2_S synthesis enzyme in the cardiovascular system [6],[7]. H_2_S has been proposed as an antioxidant due to its ability to protect against oxidative stress and to react with oxidized thiols forming hydrodisulfide [8]. In spite of its therapeutic potential, the underlying mechanisms for its beneficial effects remain unclear due essentially to the lack of reliable methods for the detection of the sulfur-containing species [8].

In the lower urinary tract, H_2_S donors produce a dual effect (contraction and/or relaxation) of smooth muscle. Thus, in rat bladder detrusor, the H_2_S donor NaHS induces contraction via stimulation of capsaicin-sensitive primary afferents (CSPA), leading to release of tachykinins, such as substance P or neurokinin A [9],[10] whereas in bladder outflow region H_2_S produces smooth muscle relaxation. In fact, in the pig bladder neck, H_2_S, synthesized by CSE, acts as a signaling molecule in the inhibitory neurotransmission, producing smooth muscle relaxation via K_ATP_ channel activation and favouring the release of the sensory neuropeptides [11],[12].

The density of the autonomic nerve fibers increases progressively from the upper ureter towards the bladder [13],[14]. In the proximal ureter, electric active pacemaker cells generate pyeloureteric rhythmicity driving adjacent smooth muscle cells thus emphasizing the role of the interstitial cells of Cajal-like cells localized at this level [15]. These cells are involved in conducting and amplifying pacemaker activity in the upper urinary tract, producing electrical slow-wave potentials favouring the propagation of ureteral peristaltic activity [15]. In the distal ureter and ureterovesical junction, in contrast, there is a rich network of autonomic nerve fibers and numerous ganglion cells that play an important role in the coordination of the ureter and bladder activity at the ureterovesical junction [13],[14],[16]. Thus, spontaneous peristaltic contractions of the upper ureter are initiated by a pacemarker activity at the renal pelvis and sustained essentially via myogenic mechanisms, whereas distal ureter activity is mainly regulated by autonomic nervous system. In fact, an NO dependent, as well as a neurogenic component of unknown nature has also been reported in the non-adrenergic, non-cholinergic (NANC) inhibitory transmission to the intravesical ureter [16],[17]. Knowledge of the mechanisms involved in the distal ureter smooth muscle relaxation is essential to provide useful therapeutic agents in the treatment of obstructive ureteral pathology produced by embedded calculi at the ureterovesical junction and in the vesico-ureteral reflux [16],[18].

H_2_S has recently been identified as a powerful inhibitory neurotransmitter in the bladder base [11]. There are no available data, however, about the H_2_S role in the distal ureter neurogenic relaxation. Therefore, the current study investigated the involvement of H_2_S in the inhibitory neurotransmission to the pig intravesical ureter.

## Materials and Methods

Adult pigs of either sex with no lesions in their urinary tract were selected from the Matadero Madrid Norte slaughterhouse. Urinary bladders with attached ureters were removed immediately after the animals were killed, and kept in chilled (4°C) physiological saline solution (PSS). The protocol was carried out in the following 24 h. The adjacent connective and fatty tissues were carefully removed, and longitudinal preparations (4–6 mm long and 2–3 mm wide) of the intravesical ureter were dissected from the bladder [19].

### Western Blot

Intravesical ureter muscle was homogenized in lysis buffer containing 10 mM Tris-HCl (pH 7.4), 1% SDS, 1 mM sodium vanadate and 0.01% protease inhibitor cocktail (all from Sigma-Aldrich, St Louis, MO, USA). 50 µg protein were separated in a 15% polyacrylamide gel (SDS-PAGE) and transferred to a polyvinylidene fluoride (PVDF) membrane (Bio-Rad). All membranes were blocked by 5% non-fat dry milk for 1 h at room temperature. For immunodetection, membranes were incubated overnight at 4°C with rabbit anti-CSE or anti-CBS (1∶1000 dilution, from Aviva Systems Biology, San Diego, USA) and mouse anti-β-actin (1∶20000 dilution, from Santa Cruz Biotechnology Heidelberg, Germany) antibodies. Membranes were then washed in 0.05% Tween-20, incubated with HRP-conjugated secondary antibodies (Alexa Fluor 594 goat-antirabbit, 1∶200 dilution, from Invitrogen, Paisley, UK) to detect CSE and CBS, for 1h at room temperature, and then washed and visualized by chemiluminescence (ECL advance-kit, GE Healthcare). Bands for CSE and CBS were normalized to those of β-actin. CSE and CBS expression in urinary bladder neck membranes were included as positive controls.

### Immunohistochemistry

Intravesical ureter segments were fixed in 4% paraformaldehyde in 0.1 M phosphate buffer, pH 7.4 (PB), for 2 to 4 h at 4°C, and subsequently placed in 30% sucrose in PB for cryoprotection. The tissue was embedded and frozen in OCT compound (Sakura Finetek, Europe BV), and stored at −80°C.Transversal sections 5 µm thick were obtained by means of a cryostat and preincubated in 10% normal goat serum in PB containing 0.3% Triton-X-100, for 2–3 h. Then, sections were incubated with either rabbit anti-CSE or anti-CBS antibodies at 4–8 µg/ml final concentration plus a mouse anti-protein gene product 9.5 (anti-PGP 9.5 from Abcam, Cambridge, UK), as neuronal marker, diluted 1∶50, during 48 h at 4°C, washed and reacted with the secondary antibodies Alexa Fluor 594 goat-antirabbit (1∶200 dilution) to detect CSE and CBS, and Alexa Fluor 488 goat-antimouse (1∶200 dilution from Invitrogen, Paisley, UK), to detect PGP 9.5, for 2 h at room temperature. The slides were covered with a specific mounting medium with DAPI (Invitrogen), which stains all cell nuclei. Observations were made with a fluorescence microscope (Olympus IX51). No immunoreactivity could be detected in sections incubated in the absence of the primary antiserum [12].

### Endogenous H_2_S measurement

H_2_S endogenous production was measured in intravesical ureter strips following the method previously described in the rat colon [20]. Briefly, the tissue was placed in a sealed polypropylene vial containing a Krebs incubation solution with 10 mM L-cysteine, 2 mM pyridoxal 5′-phosphate, 100 mM potassium phosphate buffer (pH 7.4), in the absence or in the presence of L-NOARG (100 µM), PPG (1 mM) and GYY4137 (10 µM), NO synthase (NOS) and CSE inhibitors and H_2_S donor, respectively, which was connected to a 2 ml second vial containing 0.5 ml of 1% (w/v) zinc acetate. A gas mixture of 95% O_2_ and 5% CO_2_ was bubbled from the bottom of the first vial through the incubation solution. The reaction was started by transferring the vials from ice to a water bath at 37°C. The H_2_S produced in the incubation chamber was then bubbled through the zinc acetate solution and trapped as zinc sulphide. The reaction was stopped at 30 min by injecting 0.5 ml of 50% (w/v) trichloroacetic acid into the incubation solution. Air flow was allowed to continue by an additional 30 min period, to ensure complete trapping of H_2_S in the zinc acetate solution. The content of the second vial was transferred to test tubes containing 3.5 ml of de-ionized water, 0.4 ml of N, N-dimethyl-p-phenylenediamine sulphate (20 mM) in HCl (7.2 M) and 0.4 ml of FeCl_3_ (30 mM) in HCl (1.2 M), for performing the methylene blue assay. The absorbance at 670 nm of the resulting solution was measured 20 min later by spectrophotometry (ELx800 microplate reader, Izasa). H_2_S concentration was calculated against a calibration curve of the standard NaHS solutions.

### Myographs for isometric force recordings

The intravesical ureter strips were suspended horizontally with one end connected to an isometric transducer and the other one to a micrometer screw, which regulates the tension applied to the preparations, in a myograph (DMT 820MS) containing PSS gassed with 5% CO_2_ in O_2_, giving a final pH of 7.4. Stretching of 2 g was applied to the preparations and they were allowed to equilibrate for 60 min.

The contractile ability of the strips was determined by exposing them to a 124 mM potassium PSS. In electrical field stimulation (EFS) experiments, noradrenergic neurotransmission and muscarinic receptors were blocked by pre-incubation with guanethidine (10 µM) and atropine (0.1 µM) for 1 h, replacing the solution every 20 min, and these drugs were present throughout the experiment. In strips precontracted with 0.1 µM U46619, a thromboxane A_2_ receptor agonist, EFS was performed by delivering rectangular pulses (1 ms duration, 0.5–16 Hz, 20 s trains, with constant current output adjusted to 75 mA), at 4 min intervals, from a Cibertec CS20 stimulator (Barcelona, Spain). These EFS parameters have previously been used to elicit neurogenic relaxations in the intravesical ureter [16]. A first control response curve to EFS or to the H_2_S donor *P*-(4-methoxyphenyl)-*P*-4-morpholinylphosphinodithioic acid (GYY4137, 0.1 nM- 30 µM) addition was obtained. The bath solution was then changed every 15 min for a period of 90 min, the preparations were incubated with the specific treatments for 30 min, and then a second relaxation curve was constructed. The concentration of the agents used was chosen on the basis of previous studies [11],[12]. Control curves were run in parallel.

To desensitize capsaicin-sensitive primary afferents (CSPA), strips were pre-incubated in 10 µM capsaicin for 1 h, replacing the solution every 20 min, and then experiments were conducted in the continuous presence of capsaicin [21].

#### Drugs and solutions

The following drugs were used: (2E)-N-(2, 3-dihydro-1, 4-benzodioxin-6-yl)-3-[4-(1,1-dimethylethyl)phenyl]-2-propenamide (AMG9810), atropine, and DL-propargylglycine (PPG), guanethidine, indomethacin, N^G^-nitro-L-arginine (L-NOARG) and O-(carboxymethyl)hydroxylamine (AOAA), NaHS, L-cysteine, pyridoxal 5′-phosphate, zinc acetate trichloroacetic acid, N,N-dimethyl-p-phenylenediamine sulphate, HCl, FeCl_3_ all from Sigma (St Louis, MO, USA). Calcitonin gene-related peptide 8-37 (CGRP_8-37_), capsaicin, glibenclamide, P-(4-methoxyphenyl)-P-4-morpholinylphosphinedithioic acid (GYY4137), 2-(1,3-dimethyl-2,6-dioxo-1,2,3,6-tetrahydro-7H-purin-7-yl)-N-(4-isopropyl phenyl)acetamide (HC030031), (9R,10S,12S)-2,3,9,10,11,12-hexahydro-10-hydroxy-9-methyl-1-oxo-9,12-epoxy-1H-diindolo[1,2,3-fg:3',2',1'-kl]pyrrolo[3,4-][1],[6]benzodiazocine -10-carboxylic acid (KT5720), 1H-[1],[2],[4]-oxadiazolo[4,3-a]quinoxalin-1-one (ODQ), pituitary adenylyl cyclase-activating polypeptide 6-38 (PACAP_6-38_) and 9,11-dideoxy-9a,11a-methanoepoxy prostaglandin F_2α_ (U46619) from Tocris (Bristol, UK). AMG9810, AOAA, CGRP_8-37_, PPG, glibenclamide, GYY4137, HC030031, KT5720, ODQ and PACAP_6-38_ were dissolved in dimethylsulphoxide. Indomethacin and U46619 were dissolved in ethanol. The other drugs were dissolved in distilled water. The solvents used had no effect on the contractility of the bladder neck preparations.

The composition of PSS was (mM): NaCl 119, KCl 4.6, MgCl_2_ 1.2, NaHCO_3_ 24.9, glucose 11, CaCl_2_ 1.5, KH_2_PO_4_ 1.2, ethylenediamine tetraacetic acid (EDTA) 0.027. The solution was maintained at 37°C and continuously gassed with 95% O_2_ and 5% CO_2_ to maintain pH at 7.4. K^+^-enriched PSS was PSS in which NaCl was exchanged for KCl on an equimolar basis.

#### Calculations and Statistics

Sensitivity to GYY4137 is expressed in terms of pD_2_, where pD_2_ =  -log EC_50_ and EC_50_ is the agonist concentration needed to produce half-maximal response. pD_2_ was estimated by computerized non-linear regression analysis (GraphPad Prism, USA). Results are expressed as a percentage reversal of U46619- or KPSS-induced contraction, and represent the mean ± s.e.m. of *n* (number of preparations, 1-2 strips per animal). Differences were analyzed by Student's *t*-test for paired observations and by analysis of variance and *a posteriori* Bonferroni method for multiple comparisons. The differences were considered significant with a probability level of *P*<0.05. *P* values are shown in the Figure legends.

## Results

### Expression of CSE

**Figure 1:**
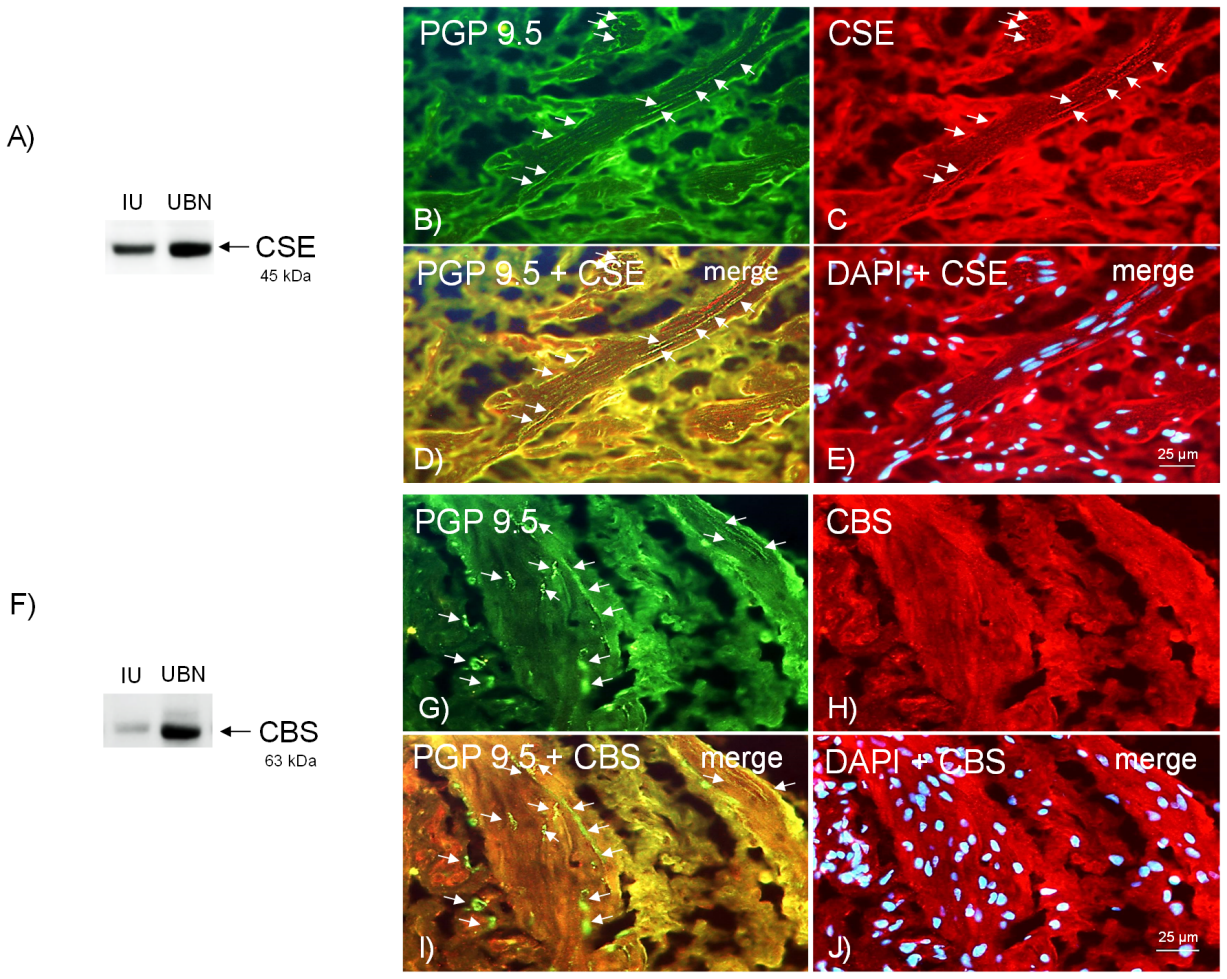
Expression of CSE protein within nerve fibers distributed among pig intravesical ureter smooth muscle bundles. (A, F) Western blot of pig intravesical ureter (IU) membranes from smooth muscle incubated with cystathionine γ-lyase (CSE) (A) and cystathionine β-synthase (CBS) (F) antibodies. Samples treated with a CSE antibody show a 45 kDa major band, thus suggesting CSE protein expression in intravesical ureter smooth muscle, whereas that CBS, however, was not consistently detected. Immunohistochemical labelling of CSE and CBS in urinary bladder neck (UBN) membranes are showed as positive controls. (B–E) Intravesical ureter immunohistochemical staining demonstrating the existence of a rich CSE-immunoreactive innervation. (B) Overall innervation of the intravesical ureter, visualized using the general nerve marker PGP 9.5 (green colour). (C) CSE immunofluorescence of the intravesical ureter shows immunopositive fibers (red colour), running parallel to the smooth muscle bundles, in the same fields as B. (D) Immunofluorescence double labelling for PGP 9.5 and CSE in the smooth muscle, showing colocalization within nerve terminals (arrows, yellow colour). (E) The cell nuclei were counterstained using DAPI (blue colour). (G–J) Immunofluorescence double staining for PGP 9.5 and CBS demonstrating the lack of a CBS-immunoreactive innervation in intravesical ureter (H). Scale bar indicates 25 µm.

By western blot, a CSE antibody recognized a band of approximately 45 kDa, which corresponded to the expected molecular weight, suggesting CSE protein expression in intravesical ureter smooth muscle (**Fig. 1A**) (n = 4 from 4 pigs). CSE and CBS expression in the intravesical ureter was also investigated by using CSE and CBS selective antibodies combined with the neuronal marker PGP 9.5. CSE immunoreactivity was observed colocalized with the neuronal marker PGP 9.5 within nerve fibers widely distributed in the smooth muscle layer running parallel to the smooth muscle bundles (**Fig. 1B–E**) (n = 5 from 5 pigs), and around the small arteries supplying the intravesical ureter (data not shown). CBS expression was not consistently detected in intravesical ureter membranes (**Fig. 1F–J**).

### Functional studies

Urothelium-denuded strips of pig intravesical ureter were allowed to equilibrate to a passive tension of 1.5±0.1 g (n = 75 preparations from 47 pigs). U46619 (0.1 µM) induced a sustained contraction above basal tension of 1.7±0.1 g (n = 75).

#### Relaxations to EFS and GYY4137

Under NANC conditions, EFS (0.5–16 Hz) evoked reproducible frequency-dependent relaxations (maximal relaxation at 16 Hz of 75±7% of the U44619-induced contraction, n = 12 from 9 pigs). The H_2_S donor GYY4137 (0.1 nM–30 µM) induced potent concentration-dependent relaxations (pD_2_ and Emax values of 7.7±0.1 and 81±7%, n = 12 from 9 pigs), which were not changed as a consequence of urothelium mechanical removal.

#### Effect of CSE and CBS blockade in the absence or presence of NOS inhibitor on EFS and GYY4137 relaxations

**Figure 2:**
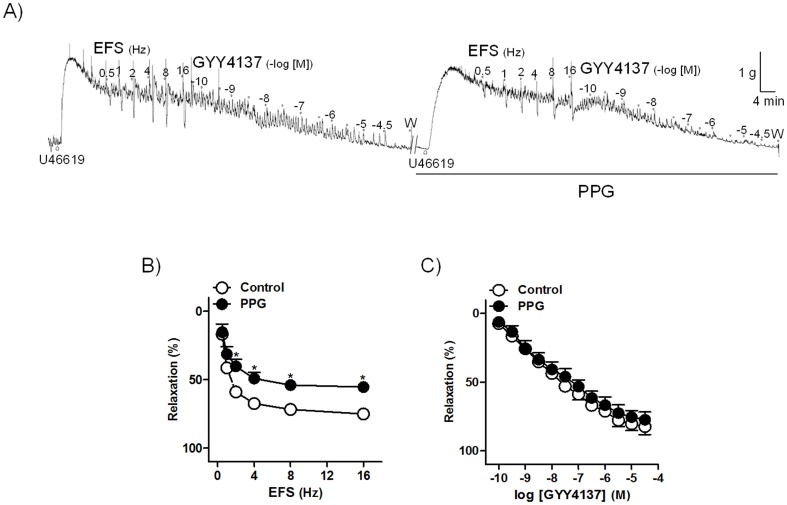
Involvement of H_2_S, synthesized by CSE, in the inhibitory neurotransmission to the intravesical ureter. (A) Isometric force recordings showing the relaxations evoked by electrical field stimulation (EFS, 1 ms duration, 0.5–16 Hz, 20 s trains) and GYY4137 (0.1 nM–30 µM), in the absence or presence of DL-propargylglycine (PPG, 1 mM), cystathionine γ-lyase inhibitor, on 0.1 µM U46619-precontracted pig intravesical ureter strips treated with guanethidine (10 µM) and atropine (0.1 µM). Vertical bar shows tension in g and horizontal bar time in min. W: wash. (B, C) Frequency- and concentration-response relaxation curves to EFS (B) and GYY4137 (C) in the absence (control, open circles) or in the presence (closed circles) of PPG. Results are expressed as a percentage reversal of the U46619-induced contraction and represent mean±s.e.m. of 8 preparations from 4 pigs. ^*^ *P*<0.05, versus control (paired *t*-test).

**Figure 3:**
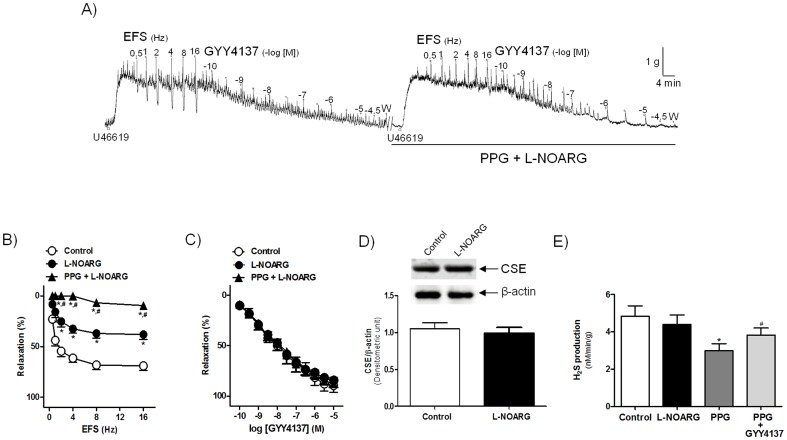
H_2_S and NO are involved in the NANC neurogenic relaxations to the intravesical ureter. (A) Isometric force recordings showing the relaxations evoked by electrical field stimulation (EFS, 1 ms duration, 0.5–16 Hz, 20 s trains) and GYY4137 (0.1 nM–30 µM), in the absence or presence of DL-propargylglycine (PPG, 1 mM) plus N^G^-nitro-L-arginine (L-NOARG, 100 µM), inhibitors of, respectively, cystathionine γ-lyase (CSE) and nitric oxide synthase, on 0.1 µM U46619-precontracted pig intravesical ureter strips treated with guanethidine (10 µM) and atropine (0.1 µM). Vertical bar shows tension in g and horizontal bar time in min. W: wash. (B, C) Frequency- and concentration-response relaxation curves to EFS (B) and GYY4137 (C) in the absence (control, open circles) or in the presence of L-NOARG (closed circles) and PPG plus L-NOARG (closed triangles). Results are expressed as a percentage reversal of the U46619-induced contraction and represent mean±s.e.m. of 7 preparations from 4 pigs. ^*,#^ *P*<0.05, versus control and L-NOARG value, respectively (analysis of variance followed by Bonferroni method). (D) Western blot intravesical ureter membranes from smooth muscle incubated with a CSE antibody in the absence and the presence of L-NOARG (100 µM). Protein levels were normalized to β-actin. Bars represent mean±s.e.m. of 4 preparations from 4 pigs (E) Level of H_2_S generated in the absence or presence of L-NOARG (100 µM), PPG (1 mM) and PPG plus GYY4137 (10 µM). Results represent mean±s.e.m. of 8 preparations from 8 pigs.^ *,#^ *P*<0.05, versus control and PPG value, respectively (analysis of variance followed by Bonferroni method).

To assess whether H_2_S plays a role in the inhibitory neurotransmission of the intravesical ureter, ureteral preparations were treated with PPG and AOAA, inhibitors of, respectively, CSE and CBS. PPG (1 mM) reduced EFS-induced relaxations (**Fig. 2A and B**), whereas AOAA (1 mM) failed to modify these responses (**Table 1**). Pretreatment with L-NOARG (100 µM) reduced the EFS relaxations (**Fig. 3B**). Incubation of ureteral strips with PPG along with L-NOARG greatly reduced the EFS responses (13% of control value at 16 Hz frequency) (**Fig. 3A and B**). Treatment with PPG (**Fig. 2C**), L-NOARG (**Fig. 3C**), PPG plus L-NOARG (**Fig. 3C**), or AOAA (**Table 2**) failed to modify GYY4137 relaxations. All these results suggest that H_2_S produced by CSE acting in concert with NO is responsible for the EFS induced relaxation of the intravesical ureter under NANC conditions.

**Table 1:** Effects of inhibitors of CBS, guanylyl cyclase, COX and PKA on relaxations induced by electrical field stimulation (EFS, 0.5–16 Hz) in the pig intravesical ureter.

				EFS (Hz)			
	*n*	*0.5*	*1*	*2*	*4*	*8*	*16*
Control	9	20±4	48±4	65±3	77±4	84±4	85±4
AOAA (1 mM)	9	24±4	53±4	69±3	78±2	85±3	85±3
Control	7	25±4	43±2	53±4	64±4	73±5	76±5
ODQ (5 µM)	7	4±2^*^	18±4^*^	27±6^*^	39±6^*^	52±5^*^	55±4^*^
Control	6	29±2	50±3	70±2	78±2	82±3	86±4
Indomethacin (3 µM)	6	32±6	51±5	68±2	76±3	81±3	84±3
Control	6	19±3	42±4	65±3	76±3	81±3	82±2
KT5720 (3 µM)	6	18±4	39±5	63±3	75±3	79±3	79±3

Results are expressed as a percentage reversal of the 0.1 µM U46619-induced contraction and represent the mean±s.e.m. of *n* preparations from 4-5 pigs. *P<0.05 versus control (paired *t*-test).

**Table 2:** Effects of inhibitors of CBS, guanylyl cyclase, COX and PKA on relaxations evoked by the H_2_S donor GYY4137 (0.1 nM–30 µM).

		GYY4137	
	*n*	*pD_2_*	*Emax (%)*
Control	9	8.7±0.1	91±5
AOAA (1 mM)	9	8.6±0.1	88±5
Control	7	8.8±0.2	94±2
ODQ (5 µM)	7	8.8±0.1	92±3
Control	6	8.3±0.1	99±1
Indomethacin (3 µM)	6	8.2±0.1	98±1
Control	6	8.4±0.2	98±1
KT5720 (3 µM)	6	8.4±0.1	98±1

Results represent the mean±s.e.m. of *n* preparations from 4–5 pigs. *Emax* is the maximal relaxation, expressed as a percentage reversal of the 0.1 µM U46619-induced contraction, obtained for each drug. pD_2_  =  -log EC_50_, where EC_50_ is the concentration of agonist producing 50% of the *Emax*.

#### Effect of NOS and CSE inhibition and of the H_2_S donor GYY4137 on endogenous H_2_S production

CSE protein expression (n = 4 from 4 pigs) (**Fig. 3D**) and endogenous H_2_S production (n = 8 from 7 pigs) (**Fig. 3E**) in intravesical ureter smooth muscle was not changed by pretreatment with the NOS inhibitor, L-NOARG (100 µM) (4.8±0.5 nM.min-1.g^−1^ and 4.4±0.5 nM.min^−1^.g^−1^, in the absence or presence of L-NOARG (*P*>0.05, versus control value, analysis of variance followed by Bonferroni method). The generated H_2_S level, however, was reduced by CSE blockade with PPG (1 mM) (2.9±0.3 nM.min^−1^.g^−1*^, n = 8) and restored by addition of the H_2_S donor, GYY4137 (10 µM) (3.8±0.5 nM.min^−1^.g^−1#^, n = 8) (^*,#^ *P*<0.05, versus control and PPG value, respectively, analysis of variance followed by Bonferroni method) (**Fig. 3E**).

#### Effect of soluble guanylyl cyclase, COX, PKA and K_ATP_ channel blockade on EFS and GYY4137 relaxations

The soluble guanylyl cyclase inhibitor ODQ (5 µM) reduced the EFS relaxations (**Table 1**) and failed to modify the GYY4137 responses (**Table 2**). Moreover, indomethacin (3 µM) and KT5720 (3 µM), blockers of, respectively, COX and PKA, did not change EFS (**Table 1**) or GYY4137 (**Table 2**) relaxations.

**Figure 4:**
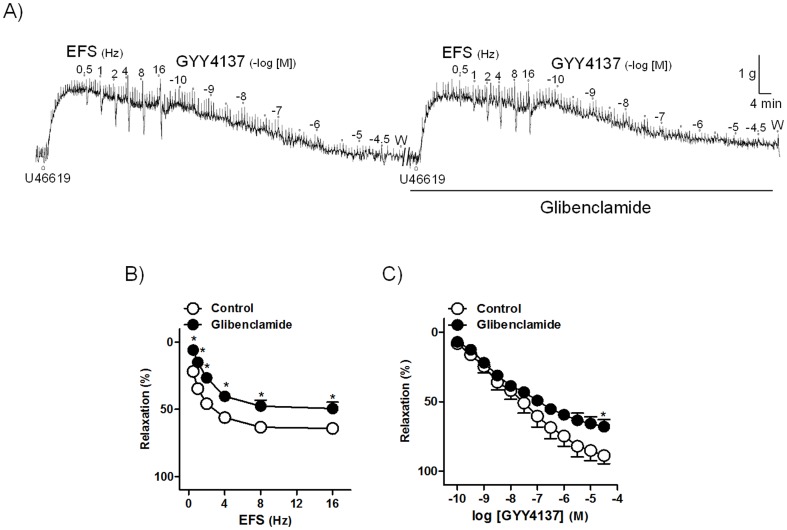
K_ATP_ channels are involved in the H_2_S relaxations. (A) Isometric force recordings showing the relaxations evoked by electrical field stimulation (EFS, 1 ms duration, 0.5–16 Hz, 20 s trains) and GYY4137 (0.1 nM–30 µM), in the absence or presence of glibenclamide (1 µM), a K_ATP_ channel inhibitor, on 0.1 µM U46619-precontracted pig intravesical ureter strips treated with guanethidine (10 µM) and atropine (0.1 µM). Vertical bar shows tension in g and horizontal bar time in min. W: wash. (B, C) Frequency- and concentration-response relaxation curves to EFS (B) and GYY4137 (C) in the absence (control, open circles) or in the presence (closed circles) of glibenclamide. Results are expressed as a reversal percentage of the U46619-induced contraction and represent mean±s.e.m. of 8 preparations from 5 pigs.^ *^ *P*<0.05, versus control (paired *t*-test).

Raising extracellular K^+^ to 80 mM induced a sustained tone of 1.7±0.1 g (n = 6). GYY4137 induced concentration-dependent relaxations on 80 mM K^+^ PSS-precontracted strips which were lower than those obtained on 0.1 µM U46619-contracted preparations (pD_2_ and Emax values of 7.8±0.1 and 83±8% and 7.7±0.1 and 63±7%^*^, in 0.1 µM U46619- or 80 mM K^+^ PSS-precontracted strips, respectively, ^*^ *P<*0.05 versus control, paired *t*-test, n = 7 from 4 pigs). Glibenclamide (1 µM), a K_ATP_ channel inhibitor, reduced both EFS and GYY4137 relaxations (**Fig. 4**), thus indicating a K_ATP_ channel involvement in the H_2_S relaxant responses.

#### Effect of capsaicin-sensitive primary afferent desensitization and of TRPA_1_ and TRPV_1_, receptor blockade on EFS and GYY4137 relaxations

**Figure 5:**
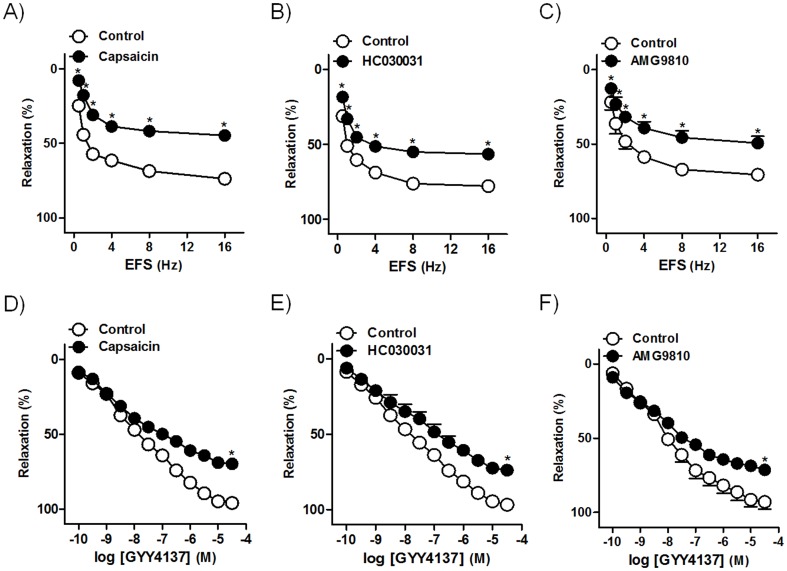
TRPA_1_ and TRPV_1_ channels from CSPA are involved in the H_2_S responses. Frequency- and concentration-response relaxation curves to electrical field stimulation (EFS, 1 ms duration, 0.5–16 Hz, 20 s trains) (A–C) and GYY4137 (0.1 nM–30 µM) (D–F) in the absence (control, open circles) or in the presence (closed circles) of capsaicin (10 µM) (A, D), HC030031 (60 µM) (B, E) and AMG9810 (10 µM) (C, F), capsaicin-sensitive primary afferent neurotoxin and TRPA_1_ and TRPV_1_ selective antagonists, respectively, on 0.1 µM U46619-precontracted pig intravesical ureter strips treated with guanethidine (10 µM) and atropine (0.1 µM). Results are expressed as a percentage reversal of the U46619-induced contraction and represent mean±s.e.m. of 7 preparations from 4 pigs.^ *^ *P*<0.05, versus control (paired *t*-test).

Capsaicin (10 µM) (**Fig. 5A and D**), a CSPA neurotoxin, as well as HC030031 (60 µM) (**Fig. 5B and E**) and AMG9810 (10 µM) (**Fig. 5C and F**), antagonists of transient receptor potential A1 (TRPA_1_) and transient receptor potential vanilloid 1 (TRPV_1_), respectively, receptors, reduced both EFS and GYY4137 responses. These data indicate that H_2_S relaxations are partly produced through TRPA_1_, TRPV_1_ and/or related ion channel activation-mediated release of inhibitory neuropeptides from CSPA.

#### Effect of VIP/PACAP and CGRP receptor blockade on EFS and GYY4137 relaxations

**Figure 6:**
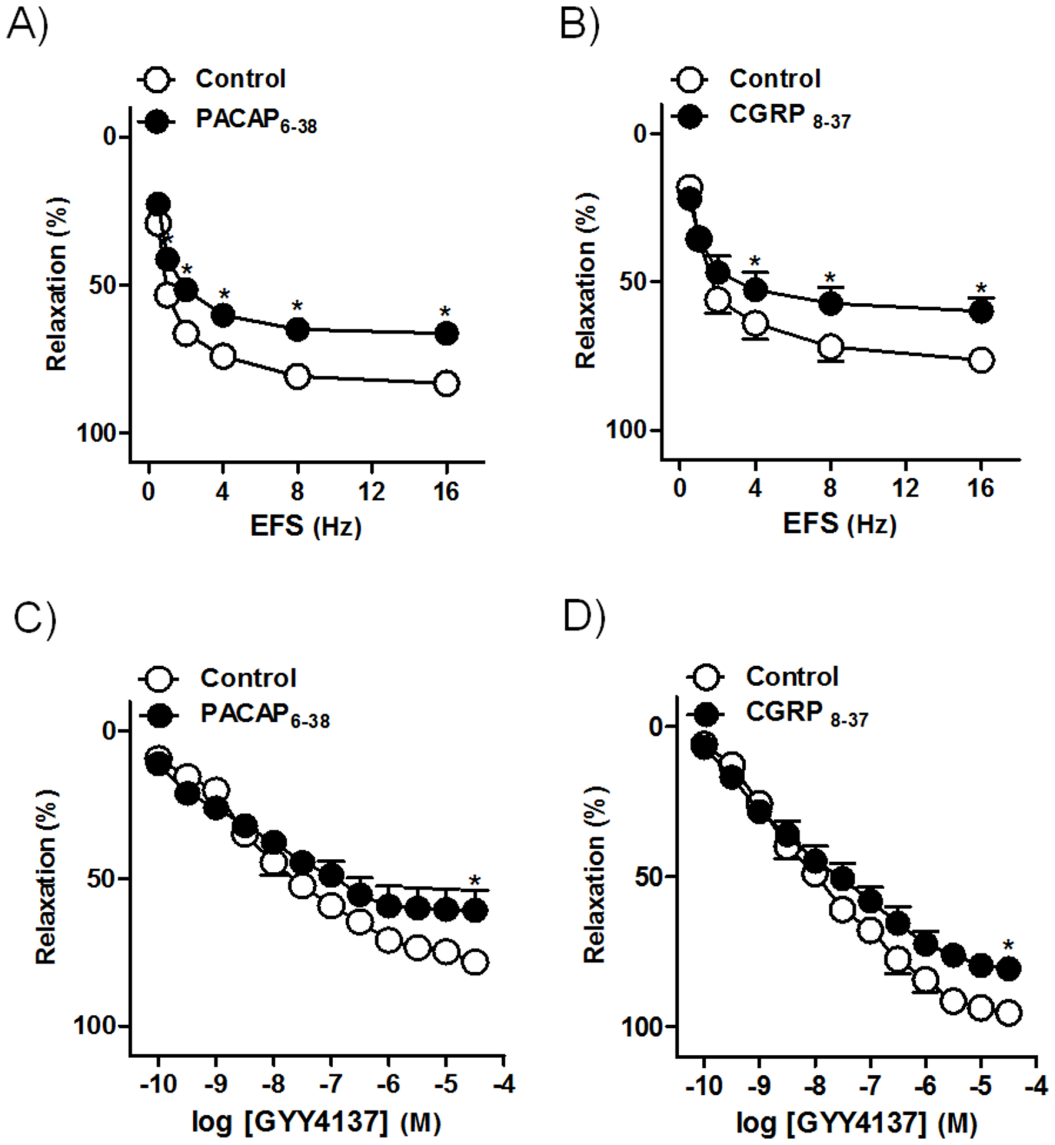
PACAP and CGRP might be involved in the H_2_S relaxations. Frequency- and concentration-response relaxation curves to electrical field stimulation (EFS, 1 ms duration, 0.5–16 Hz, 20 s trains) (A, B) and GYY4137 (0.1 nM–30 µM) (C, D) in the absence (control, open circles) or in the presence (closed circles) of PACAP_6–38_ (3 µM) (A, C) and CGRP_8–37_ (3 µM) (B, D), VIP/PACAP and CGRP receptor antagonists, respectively, on 0.1 µM U46619-precontracted pig intravesical ureter strips treated with guanethidine (10 µM) and atropine (0.1 µM). Results are expressed as a percentage reversal of the U46619-induced contraction and represent mean±s.e.m. of 7–8 preparations from 4 pigs.^ *^ *P*<0.05, versus control (paired *t*-test).

PACAP _6–38_ (3 µM) (**Fig. 6A and C**) and CGRP_8–37_ (3 µM) (**Fig. 6B and D**), antagonists of VIP/PACAP and CGRP, respectively, receptors, reduced both EFS and GYY4137 responses, thus suggesting that a part of H_2_S relaxation might be due to PACAP 38 and CGRP.

## Discussion

Our results provide morphological and functional evidence that neuronal H_2_S, synthesized by CSE, is involved in the NO-independent NANC inhibitory transmission to the pig intravesical ureter. H_2_S induces smooth muscle relaxation via K_ATP_ channel activation and also promotes the release of inhibitory neuropeptides PACAP 38 and CGRP from CSPA, through sensory nerve TRPA_1_, TRPV_1_ and/or related ion channel activation. This conclusion is supported by the following observations: (1) The presence of CSE within nerve fibers widely distributed in the smooth muscle layer of the intravesical ureter. (2) The neurogenic relaxation elicited by EFS was inhibited by PPG. (3) EFS and GYY4137 responses were reduced by blockade of K_ATP_ channels, desensitization of CSPA and inhibition of TRPA_1_ and TRPV_1_ channels and of PACAP and CGRP receptors.

In the pig intravesical ureter, only CSE expression was consistently observed. Western blot assays showed a band compatible with that expected for CSE in the muscular layer, and immunostaining of ureteral samples revealed a labeling for CSE protein within nerve fibers widely distributed among smooth muscle bundles. The high density and the distribution of CSE immunoreactivity observed in the intravesical ureter agree with that found in pig bladder neck [12]. In the current study, the existence of CSE-immunoreactive elements around small arteries also suggests a role for H_2_S in the regulation of intravesical ureter blood flow, thus supporting an important role for H_2_S in vascular tone modulation [22].

NO-dependent and independent NANC neurogenic relaxations in the intravesical ureter have previously been reported [16],[17]. In the current investigation, isometric force recording experiments showed that the CSE inhibitor PPG reduced the EFS-elicited neurogenic relaxations, whereas that the CBS inhibitor AOAA failed to modify these responses, reinforcing the validity of observations made about the lack of CBS immunoreactivity in the intravesical ureteral wall. These results, together with the reduction of endogenous H_2_S production elicited by PPG and its recovery in response to GYY4137, clearly indicate that neuronally-released endogenous H_2_S synthesized by CSE is responsible for a considerable part of the NANC inhibitory transmission to the intravesical ureter. The fact that incubation with the NO synthase inhibitor L-NOARG plus PPG abolished the EFS relaxations indicates that, in addition with NO, H_2_S plays a key role in ureteral inhibitory neurotransmission, and might therefore directly be involved in the regulatory mechanisms of the smooth muscle tone, thus reducing the ureteral resistance during bladder filling. In the intravesical ureter, in addition to the predominant longitudinal smooth muscle fibers, circular and helical fibers have also been described [13],[14], so that other mechanisms might be involved in the regulation of the ureteral smooth muscle contractility. In the current study, an effect of GYY4137 on the amplitude and frequency of the U46619 contractions was not consistently observed. Further in vivo studies would be necessary to assess the changes induced by H_2_S in the distal ureter urodynamic parameters.

NO has been proposed as an inducer or as a molecular switch for endogenous H_2_S production for regulating of vascular smooth muscle tension [2]. In the current study, the fact that endogenous H_2_S production rate was not modified under conditions of NOS blockade suggests the involvement of a NO-independent pathway in the intravesical ureter endogenous H_2_S generation. These results agree with those obtained in bladder neck, where both H_2_S [12] and NO [23] neuronal pathways promote smooth muscle relaxation. Current results showing the mediation of H_2_S, together with NO, in the intravesical ureter neurogenic relaxation reinforces the role of the autonomic nervous system in the regulation to the distal ureter tension in contrast with the myogenic electrical activity characteristic of the pyeloureteral segments [13],[14],[16],[17].

In our study, GYY4137, a donor which in the cardiovascular system slowly releases H_2_S, both in vivo and in vitro [24], produced a potent relaxation (pD_2_ value of 7.7), slow in onset and sustained, which was similar to that previously obtained in bladder neck [12] indicating an essential role for H_2_S in the ureteral smooth muscle relaxation. The fact that PPG failed to modify the GYY4137 relaxations may be explained on the basis that PPG is an inhibitor of the endogenous H_2_S synthesis enzyme CSE, and therefore does not seem probable that it can reduce the responses to the exogenously-added H_2_S donors. Current results agree with those obtained in bladder neck, where CSE selective blockade did not change the GYY4137 responses [12]. Urothelium mechanical removal, as well as pretreatment with the NO enzyme synthesis inhibitor L-NOARG did not change the GYY4137 relaxations, thus suggesting that H_2_S produces smooth muscle relaxation via urothelium- or NO-independent mechanisms.

Like neuronal- and endothelial-NOS, CSE activity is Ca^2+^-calmodulin dependent [25] and H_2_S generated from L-cysteine by CSE exerts its biological action by sulfhydrating target proteins, process that may augment guanylyl cyclase activity, thus increasing [cGMP]_i_ and relaxing smooth muscle [26]. In the present study, relaxations to EFS were reduced by ODQ, a soluble guanylyl cyclase inhibitor. This is consistent with previous findings in the intravesical ureter showing that NO-mediated NANC neurogenic relaxation is produced, in part, via activation of guanylyl cyclase [17]. ODQ, however, failed to modify the GYY4137 responses, thus initially ruling out an involvement of the cGMP/NO-dependent mechanisms in the H_2_S relaxations. These results agree with those previously described in vascular smooth muscle, where unlike the intracellular signaling responsible for the vasodilator action induced by NO and CO, H_2_S relaxations were produced in a guanylyl cyclase activation-independent way [27].

H_2_S has previously been reported to inhibit superoxide anions formation via adenylyl cyclase-PKA pathway in pig pulmonary arterial endothelial cells [28]. In the current study, however, the lack of effect shown by the PKA inhibitor KT5720 on EFS or GYY4137 responses seems to rule out the involvement of the PKA pathway in H_2_S relaxations.

K_ATP_ channel activation mediates the H_2_S-induced relaxation in both vascular and visceral smooth muscle. Thus, K_ATP_ channel opening-mediated H_2_S responses have been described in rat aorta and mesenteric arteries [3],[5],[22] or pig bladder neck [11]. In the intravesical ureter, GYY4137 relaxations were reduced in 80 mM K^+^ PSS-precontracted strips. Extracellular [K^+^] elevation inhibits K^+^ efflux through membrane K^+^ channels, and since glibenclamide, a K_ATP_ channel inhibitor, reduced the EFS or GYY4137 responses, it seems likely that ionic conductance modifications via K_ATP_ channels are involved in H_2_S relaxations. Interestingly, this signaling pathway is also involved in the neuronal NO-mediated relaxation of the pig intravesical ureter [17].

The COX pathway is involved in bladder physiology and pathology, and several studies have demonstrated a role for COX-derived prostanoids in the neural control of bladder smooth muscle tone [11],[29],[30],[31]. In the current study, indomethacin, a COX inhibitor, failed to modify the EFS or GYY4137 relaxations, thus indicating that COX-derived prostanoids are not likely to be involved in the H_2_S responses.

H_2_S donors produce contraction of rat detrusor via release of tachykinins such as substance P or neurokinin A from CSPA, by activating non-selective cation channel TRPV_1,_ TRPA_1_ and/or related ion channels in the sensory nerves [9],[10],[32]. Sensory neuropeptides, such as pituitary adenylyl cyclase-activating polypeptide 38 (PACAP 38) relax the intravesical ureter [33]. In the pig bladder neck, the H_2_S relaxant responses are produced, in part, via PACAP 38 and calcitonin gene-related peptide (CGRP) release from CSPA [11]. For this reason, we investigated whether in the intravesical ureter, the release of sensory neuropeptides such as PACAP 38 and/or CGRP could be involved in the H_2_S relaxations. The protocol of capsaicin desensitization carried out in our investigation produces an intravesical ureter CSPA functional blockade [21]. Thus, the reduction of the EFS or GYY4137 relaxations caused by capsaicin would indicate that these responses are produced, in part, by inhibitory peptides released from CSPA. TRPA_1_ are recognized as the main target for H_2_S in sensory neurons [34]. In the current study, the inhibition produced by HC030031, a TRPA_1_ selective antagonist, on EFS or GYY4137 responses, suggests the involvement of TRPA_1_ receptors in H_2_S relaxations. Moreover, the H_2_S response reduction produced by blockade of TRPV_1_ with AMG9810 indicates the mediation of these receptors. The fact that capsaicin inhibition of the EFS or GYY4137 relaxations was higher than that exerted by HC030031 and AMG9810 suggests that in addition to the TRPA_1_ and TRPV_1_, the possible role of related ion channels located on sensory neurons. The EFS or GYY4137 response inhibition produced by VIP/PACAP and CGRP receptor blockade, suggests that H_2_S may promote intravesical ureter smooth muscle relaxation via PACAP 38 and/or CGRP release from CSPA. These results agree with those found in the pig bladder neck, where part of H_2_S relaxations are indirectly produced via inhibitory neuropeptide release from sensory nerves [11].

H_2_S donors have been proposed as helpful therapeutic tools for unilateral ureteric obstruction-induced renal damage by attenuating fibrosis, oxidative stress and inflammation [35]. Neurogenic mechanisms play an essential role in distal ureteral motility. In fact, intravesical ureter efferent and afferent innervation, including cholinergic, adrenergic and NANC components, is much dense than that in the upper ureter in humans [36],[37]. Most urinary stones are frequently located distally [38], therefore a better understanding of the neurogenic mechanisms involved in distal ureteral smooth muscle relaxation could lead to the discovery of new drugs useful in relieving ureteral colic, facilitating spontaneous stone passage, relieving symptoms or preparing the ureter for ureteroscopy. Our lab previously demonstrated the involvement of NO and an unknown nature mediator/s in the intravesical ureter neurogenic relaxation [16] [17]. Current results show that, beside NO, H_2_S is responsible for the intravesical ureter NANC inhibitory neurotransmission, thus suggesting that H_2_S-mediated neurotransmission might be useful as a therapeutic target in the obstructive ureteral pathology and in the vesico-ureteral reflux.

**Figure 7:**
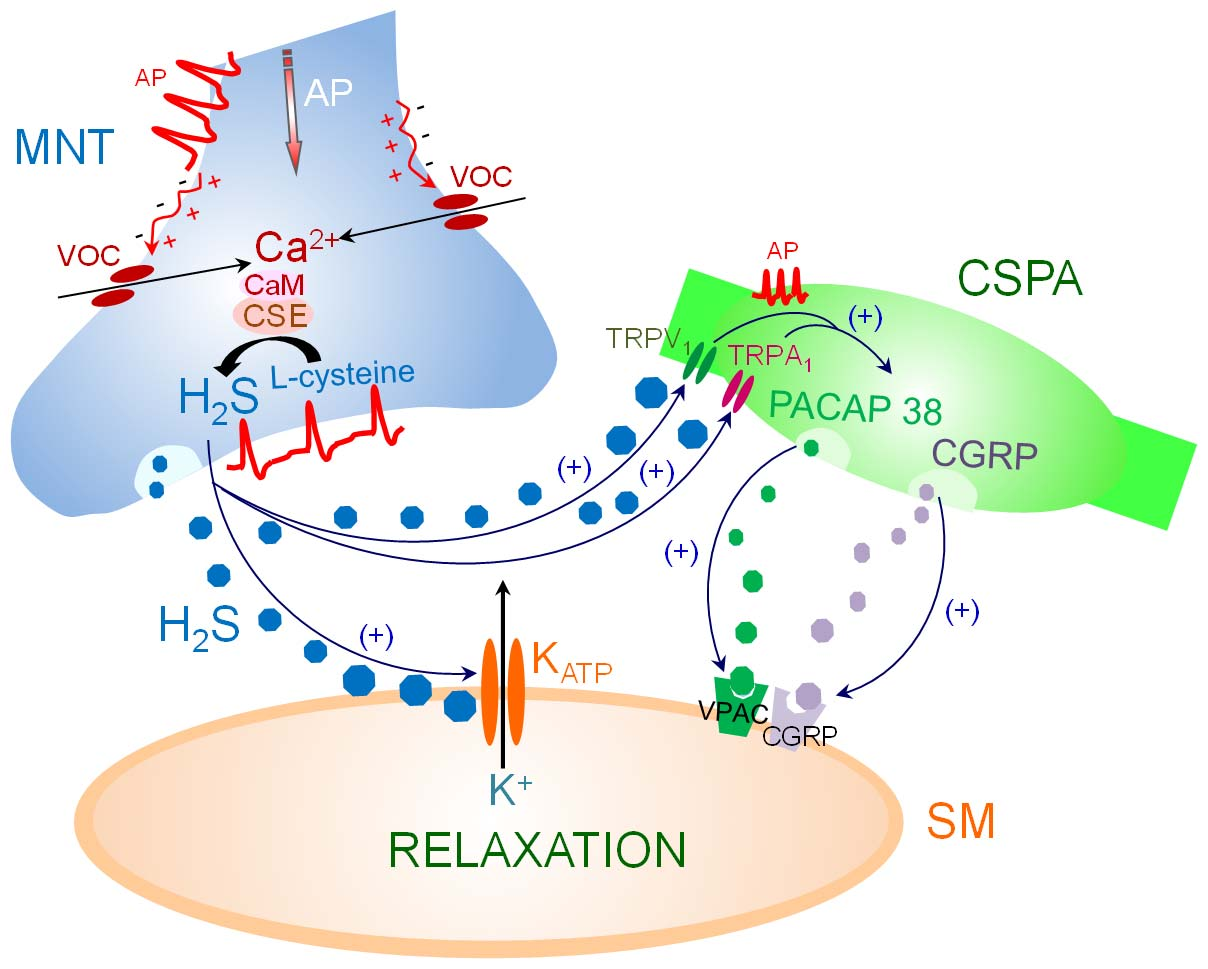
Proposed signaling for neuronal H_2_S in pig intravesical ureter. Arrival of action potentials (AP) at the motor nerve terminal (MNT) nerve ending evokes membrane depolarization and activation of voltage-gated Ca^2+^ (VOC) channels with the subsequent Ca^2+^ influx, which would stimulate neuronal cystathionine γ-lyase (CSE), through interaction with calmodulin (CaM) favouring H_2_S synthesis from L-cysteine and release from nerves. H_2_S would diffuse across the synaptic cleft producing postjunctional K_ATP_ channel activation, membrane hyperpolarization by K^+^ efflux and subsequent smooth muscle (SM) relaxation. In addition, H_2_S might promote TRPA_1_ and TRPV_1_ channel activation from capsaicin-sensitive primary afferent (CSPA), thus favouring the release of pituitary adenylyl cyclase-activating polypeptide 38 (PACAP 38) and calcitonin gene-related peptide (CGRP), which in turns would produce smooth muscle relaxation via activation the VIP/PACAP (VPAC) and CGRP, respectively.

In conclusion, present results suggest that H_2_S, synthesized by CSE, acts as a potent inhibitory neurotransmitter to the pig intravesical ureter through a NO-independent mechanism, producing smooth muscle relaxation via K_ATP_ channel activation. H_2_S also promotes the release of PACAP 38 and CGRP from CSPA through activation of TRPA_1_, TRPV_1_ and/or related ion channels in the sensory nerves (**Fig. 7**). To our knowledge this is the first study showing the involvement of H_2_S in the neurogenic relaxation of the intravesical ureter.
